# Evaluation of the Beck Anxiety Inventory in predicting generalised anxiety disorder among individuals seeking HIV testing in the Western Cape province, South Africa

**DOI:** 10.4102/sajpsychiatry.v25i0.1336

**Published:** 2019-11-27

**Authors:** Wylene L. Saal, Ashraf Kagee, Jason Bantjes

**Affiliations:** 1 Department of Psychology, Arts and Social Sciences, Stellenbosch University, Cape Town, South Africa

**Keywords:** generalised anxiety disorder, sensitivity, specificity, receiver operating curve analysis, HIV, Western Cape, South Africa

## Abstract

**Background:**

Routine anxiety screening is needed among HIV test seekers, given the lack of health-care professionals with the ability to identify individuals with generalised anxiety.

**Aim:**

The aim of this study was to determine the effectiveness of the Beck anxiety inventory (BAI) in predicting caseness for generalised anxiety disorder (GAD) among persons seeking HIV testing, using the structured clinical interview for the DSM-5 (SCID-5) as the gold standard.

**Setting:**

Five HIV testing sites in the Western Cape region of South Africa.

**Method:**

We recruited 500 persons seeking HIV testing from five non-medical testing sites in the Western Cape, South Africa. We used receiver operating curve analysis to determine the optimal cut-off point on the BAI to discriminate between GAD caseness and non-caseness.

**Results:**

3.4% of the sample met the DSM-5 criteria for a diagnosis of GAD. Using an optimal cut-off point of 21.5, the sensitivity and specificity of the BAI were 82% and 80%, respectively. The positive predictive value was 13%, while the negative predictive value was 99%.

**Conclusion:**

Our data suggest that while the BAI may be used to screen for GAD, it is likely to yield a high number of false positives. A two-tiered method may be useful to mitigate against case over-identification. Thus, in a public health setting, persons screening positive on the BAI should receive a diagnostic interview to determine whether they are true cases for GAD. Within resource-constrained communities in South Africa, referral trajectories should be integrated with routine screening and HIV testing.

## Introduction

Generalised anxiety disorder (GAD) is common among people living with HIV (PLWH).^1,2,3^ GAD is highly impairing and is associated with poor quality of life and low levels of adherence to HIV treatment.^4,5^ Yet, using diagnostic interviews to identify cases of mental disorders, including GAD, is often cost and resource intensive. Moreover, there are insufficient healthcare professionals in the public health systems in resource-constrained countries such as South Africa who possess the necessary knowledge and skills required to diagnose GAD.^6^ Therefore, screening may be necessary to identify individuals with GAD in these environments. We sought to evaluate the effectiveness of the Beck anxiety inventory (BAI) in detecting GAD among people seeking HIV testing. Successful case identification will enhance the likelihood of persons who need treatment.

The ability of screening instruments to discriminate between GAD cases and non-cases is usually assessed in terms of the optimal combination of sensitivity and specificity.^7,8^ Sensitivity refers to the ability of the screening instrument to accurately detect individuals with the disorder.^7,8^ A screening instrument with poor sensitivity will miss a large number of individuals who meet the diagnostic criteria for the disorder, yielding a large number of false negative results. Specificity, on the other hand, refers to the ability of the screening instrument to accurately detect individuals without the disorder.^7,8^ A screening instrument with poor specificity will positively identify a large number of individuals who do not meet the diagnostic criteria for the disorder, yielding many false positives.

Screening instruments, compared to structured interviews, are short, easy to use and require minimal training for test administrators. The major disadvantage of screening, however, is the possibility of yielding large numbers of false positive and false negative cases.^9^ Over-identification of GAD can lead to an added financial burden on the healthcare system in resource-constrained communities, as false positive cases may be unnecessarily referred for treatment.^10^

### Generalised anxiety disorder and HIV

The prevalence of GAD among people living with HIV varies widely, depending on the methods of assessment used, that is, structured diagnostic interviews and self-report measures. For example, using the MINI international neuropsychiatric interview (MINI), 13.3% of 649 HIV-infected individuals in Zambia met the diagnostic criteria for GAD.^11^ Similarly, Adewuya et al.^1^ reported that 11.4% of 88 Nigerian HIV-infected individuals met the diagnostic criteria for GAD using the MINI. However, among 456 HIV-positive patients in South Africa, the prevalence of GAD as assessed by the MINI was 4.6%.^2^ An even lower rate of GAD of 0.4% was found among 900 HIV-positive individuals using the Composite International Diagnostic Interview (CIDI).^3^ As treatment for HIV has become more accessible in recent years, a diagnosis of HIV has become less life-threatening, which may explain the lower rates of symptoms of anxiety among HIV-infected individuals.^12^ Even though the prevalence of GAD has been documented among persons living with HIV, we were only able to find one study among individuals seeking an HIV test. In Goa, India, 1.1% of 1874 persons receiving HIV testing reported elevated symptoms of anxiety using the 7-item generalised anxiety scale (GAD-7).^13^

The aim of this study was to determine the prevalence of GAD caseness and sub-threshold anxiety symptoms among persons seeking an HIV test. We also sought to assess the effectiveness of the BAI in discriminating between those who met the criteria for GAD and those who did not. These data were collected as part of a PhD thesis.^14^

## Method

### Participants

Five hundred participants were enrolled in the study by means of convenience sampling before undergoing HIV testing at five HIV testing sites in the Western Cape region of South Africa. HIV testing is conducted under the auspices of the Western Cape Department of Health and outsourced to testing sites. Personnel at the testing site set up temporary structures in open public spaces in the community, such as the parking lots of shopping centres or taxi ranks. All persons seeking an HIV test who were conversant in English were eligible to participate in the study and non-English (*n* = 40) speaking participants were excluded from the study.

### Procedures

At the clinic reception, HIV test seekers were handed a flyer that described the study. They were then invited to meet with a researcher in a private room. Those participants who agreed were informed about the study in more detail and were invited to participate.

### Measures

#### Generalised anxiety disorder

We used the Structured Clinical Interview for the DSM-5 (SCID-5- Research Version) to determine caseness for GAD. The SCID-5 has been used as the ‘gold standard’ in several studies to ascertain the presence of clinical disorders^15,16^ and is anchored to the Diagnostic and Statistical Manual of Mental Disorders (DSM-5). The SCID was chosen as the gold standard instead of the MINI and CIDI because it adheres more strictly to the DSM 5 diagnostic criteria. Also, while the MINI is short and brief, it only provides a diagnosis at a single time point,^17,18^ whereas the SCID provides current and past diagnoses of mental health disorders,^19^ which were also assessed in this study.

We conducted a pilot study to assess whether the participants understood the questions on the SCID. The results indicated that the SCID questions would be comprehensible among the sample. Data collectors received intensive training in administration of the SCID to ensure that they kept their responses to interview items as consistent as possible and quality checks were conducted to ensure fidelity to the SCID interview schedule. The SCID questions were set up on a web-based platform and the responses were captured on a Lenovo tablet.

#### Symptoms of anxiety

The 21-item BAI assesses the severity of anxiety in patients with psychiatric disorders^20^ with a total score ranging between 0 and 63.^20,21^ Notably, the BAI assesses symptoms of anxiety in the past 2 weeks and not acute anxiety (i.e., anxiety at the time of undergoing HIV testing). The BAI has a four-point Likert-type scale which ranges from 0 (‘not at all’) to 3 (‘severely’ – I could barely stand it).^20^ A total score of between 0 and 7 on the BAI indicates minimal anxiety, 8–15 indicates mild anxiety, 16–25 indicates moderate anxiety and between 26 and 63 indicates severe anxiety.^20,21^ The BAI has consistently shown high internal consistency (e.g., Cronbach’s alpha = 0.92;^20^), including among South African samples.^22,12^ For example, the Cronbach’s alpha of a Xhosa version of the BAI was 0.92, indicating high internal consistency.^21^ In an exploratory factor analysis among 101 HIV-positive individuals in South Africa receiving ART, a single factor structure accounted for 68.7% of the variance among the items.^12^

### Ethical considerations

The study obtained ethical approval from the Stellenbosch University Health Ethics Committee (N13/05/062). An informed consent form was signed by all participants. Those participants who were found to be psychologically distressed or being diagnosed with a psychiatric disorder were referred to a nearby mental health facility for further services.

## Results

### Sample demographic characteristics

We recruited 540 participants including 40 participants who were ineligible because they could not understand English. Consequently, as can be seen in Table 1, the total sample consisted of 500 participants of which 51.6% identified as women, and 48.4% as men. The mean age of the participants was 36 years. A total of 98.8% of participants were from historically disadvantaged groups, that is, they self-identified as black or mixed race people. The majority of the sample (69.0%) were Afrikaans-speaking individuals, while 6.0% were English-speaking and 19.6% were isiXhosa-speaking individuals.

**TABLE 1:** Sample demographic characteristics.

Characteristics	*N*	%	95%CI
**Gender**			
Male	242	48.4	44.02–52.78
Female	258	51.6	47.22–55.98
**Age (years)**			
Mean	-	36	-
18–19	27	5.4	3.42–7.38
20–29	150	30.0	25.98–34.02
30–39	139	27.9	23.97–31.83
40–49	102	20.5	16.96–24.04
50–71	80	15.9	12.69–19.11
**Race**			
Black	131	26.2	22.35–30.05
Mixed race	363	72.6	68.69–76.51
White	4	0.8	0.02–1.58
Other	2	0.4	−0.15–0.95
**First language**			
Afrikaans	345	69.0	64.95–73.05
English	30	6.0	3.92–8.08
isiXhosa	98	19.6	16.12–23.08
Other	27	5.4	3.42–7.38
**Current work situation**			
Employed full-time	97	19.4	15.93–22.87
Employed part-time	106	21.2	17.62–24.78
Unemployed	233	46.6	42.23–50.97
Homemaker	11	2.2	0.91–3.49
Student	29	5.8	3.75–7.85
Disabled	7	1.4	0.37–2.43
Retired	17	3.4	1.81–4.99

*N*, sample size; %, percentage; 95% CI, 95% confidence interval.

### Generalised anxiety disorder and symptoms of anxiety

The prevalence of GAD among our sample was 3.4% (*n* = 17) while 96.6% (*n* = 483) did not have GAD. The internal consistency as measured by Cronbach’s alpha for the BAI was excellent (0.94). The mean score of 12.58 on the BAI fell in the range of minimal anxiety. As can be seen in Table 2, 78.2% of the sample reported minimal anxiety, 13.6% reported moderate anxiety and 8.2% reported severe anxiety.

**TABLE 2:** Percentage of sample in each Beck anxiety inventory category.

BAI category	*N*	%	95%CI
Mild anxiety (0–21)	391	78.20	74.58–81.82
Moderate anxiety (22–35)	68	13.60	10.6–16.6
Severe anxiety (36–63)	41	8.20	5.8–10.6

*N*, sample size; %, percentage; 95% CI, 95% confidence interval; BAI, Beck anxiety inventory.

### Receiver operating characteristic analysis for generalised anxiety disorder

**FIGURE 1:**
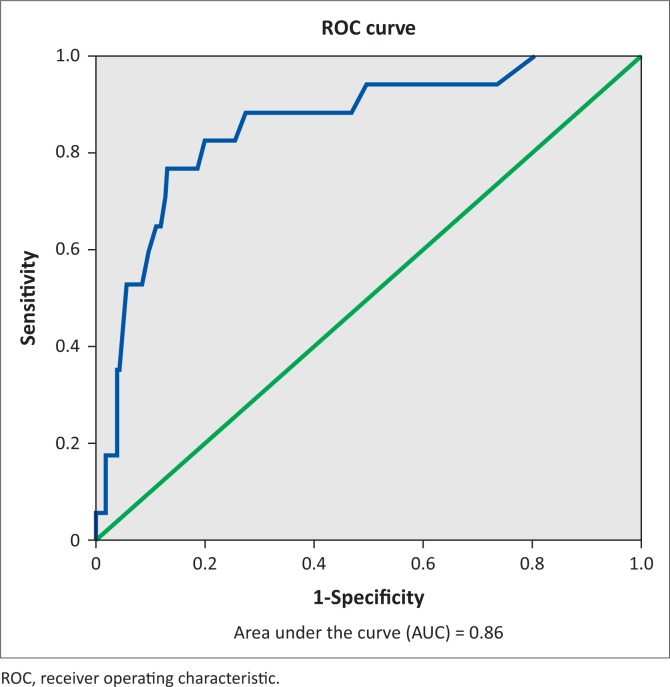
Area under the curve.

The receiver operating characteristic (ROC) curve in Figure 1 shows the performance of the BAI in correctly identifying GAD. The area under the curve (AUC) of 86% (AUC = 0.86) indicates that the BAI is moderately accurate in determining cases of GAD.

Table 3 shows the optimal cut-off point of 21.5, which yielded the optimal combination of sensitivity (0.82) and specificity (0.80).

**TABLE 3:** ROC curve coordinates of the Beck anxiety inventory using the SCID as the gold standard.

Cut-off point	Sensitivity	1-Specificity	Specificity
1.0000	1.000	1.000	0.000
0.5000	1.000	0.874	0.126
1.5000	1.000	0.805	0.195
2.5000	0.941	0.735	0.265
3.5000	0.941	0.692	0.308
4.5000	0.941	0.627	0.373
5.5000	0.941	0.590	0.410
6.5000	0.941	0.538	0.462
7.5000	0.941	0.497	0.503
8.5000	0.882	0.470	0.530
9.5000	0.882	0.435	0.565
10.5000	0.882	0.414	0.586
11.5000	0.882	0.383	0.617
12.5000	0.882	0.356	0.644
13.5000	0.882	0.335	0.665
14.5000	0.882	0.311	0.689
15.5000	0.882	0.288	0.712
16.5000	0.882	0.271	0.729
17.5000	0.824	0.253	0.747
18.5000	0.824	0.238	0.762
19.5000	0.824	0.222	0.778
20.5000	0.824	0.213	0.787

**21.5000**	**0.824**	**0.197**	**0.803**

ROC, receiver operating characteristic; BAI, Beck anxiety inventory; SCID, structured clinical interview for the DSM-5.

Note: The row in bold indicates the optimal cut-off point which yielded optimal sensitivity and specificity.

As can be seen in Table 4, 17 participants (3.4%) out of the total sample (*n* = 500) were diagnosed with GAD using the SCID. Of these 17 participants, 14 (82.4%) participants scored above 21.5 on the BAI and met the diagnostic criteria for GAD using the SCID, that is, they were true positives. However, of the 17 participants, 3 (17.6%) participants scored below 21.5 on the BAI but met the diagnostic criteria for GAD using the SCID, that is, they were false negatives.

**TABLE 4:** Two-by-two table of BAI versus structured clinical interview generalised anxiety disorder diagnosis.

BAI cut-off score	Positive (SCID)	*n*	Negative (SCID)	*n*	Total
BAI ≥ 21.5	14 (true positive)	a	97 (false positive)	c	a + c = 111
BAI < 21.5	3 (false negative)	b	386 (true negative)	d	b + d = 389

**Total**	**-**	**a + b = 17**	**-**	**c + d = 483**	**-**

*n*, sample size; BAI, Beck anxiety inventory; SCID, structured clinical interview for the DSM-5.

Conversely, 483 (96.6%) participants of the total sample were not diagnosed with GAD using the SCID. Of these 483 participants, 97 participants (20.1%) scored above 21.5 on the BAI but did not meet the diagnostic criteria for GAD using the SCID, that is they were false positives.

Of the 483 participants, 386 (80.0%) participants scored below 21.5 on the BAI and did not meet the diagnostic criteria for GAD on the SCID, that is they were true negatives.

As can be seen in Table 5, the positive predictive value (PPV) was 13.0% and the negative predictive value (NPV) was 99.2%. These data indicate a 13.0% probability that individuals who scored above 21.5 would meet the diagnostic criteria for GAD, and a 99.2% probability that individuals who scored below 21.5 would not meet the criteria for the disorder.

**TABLE 5:** Sensitivity, specificity and predictive values of the Beck anxiety inventory with reference to the optimal cut-off point of 21.5.

Test characteristics	Formula	Expression	Value	%	95% CI
Sensitivity	a/(a+b)	14/17	0.82	82.35	79.01–85.69
Specificity	d/(c+d)	386/483	0.80	80.00	76.49–83.51
Positive predictive value (PPV)	a/(a+c)	14/111	0.13	13.00	10.05–15.95
Negative predictive value (NPV)	d/(b+d)	386/389	0.99	99.23	98.46–100.00

95% CI, 95% confidence interval.

## Discussion

Among our sample, the prevalence of GAD was 3.4%. Olley, Seedat and Stein^23^ found higher GAD rates among HIV-positive individuals at both baseline (6.7%) and at 6-month follow-up (6.2%) visits, compared to the current study of 3.4%. Yet, among 900 HIV-positive South Africans the prevalence of GAD was 0.4%.^3^ The differences in prevalence rates may be attributed to the discrepancies in the measuring instruments used (i.e., MINI and CIDI), compared to the current study which used the SCID as well as the nature of the sample (i.e., persons receiving HIV testing rather than those living with HIV).

The mean total score (12.58) of the BAI fell in the range of minimal anxiety. However, a minority (13.6%) of the sample scored in the moderate to severe range, suggesting that anxiety was a concern for these individuals. For this group, it was possible that anxiety may have been due to the prospect of undergoing an HIV test, a finding that is in keeping with previous research that found elevated levels of anxiety (19.5%) among South Africans living with HIV.^12^

As a screening tool, the BAI performed well in discriminating between GAD caseness and non-caseness (AUC = 0.86), and indicated it has moderately high accuracy in identifying GAD.^24^ The cut-off point of 21.5 yielded optimal sensitivity (82.4%) and specificity (80.0%) values, indicating that this cut-off point may be suitable in discriminating between GAD cases and non-cases among HIV test seekers.

Using the cut-off point of 21.5, the BAI yielded a low PPV (13.0%) in identifying GAD, suggesting a 13.0% probability that individuals with a positive test would be diagnosed with GAD. The PPV is dependent on the prevalence estimate of the underlying condition or disorder, in this case GAD.^7,8^ As the prevalence of GAD was low in the sample, the PPV was also low. On the other hand, the NPV was 99.2%, suggesting a 99.2% probability that individuals with a negative test would be identified as not having GAD. It appears therefore that the BAI is highly successful at predicting non-caseness but less successful at predicting caseness for GAD given its low prevalence.

Although those individuals who screen positive for GAD may benefit from referral to treatment, a positive screen on its own is likely to be inadequate to yield a diagnosis of a psychiatric disorder.^10^ Given our data, it is recommended that persons who score above the cut-off point of the BAI undergo a follow-up assessment with a diagnostic interview such as the SCID to confirm a diagnosis of GAD.^25^

### Limitations of the study

A limitation of this study is that we recruited participants from HIV testing sites in the Western Cape only, which limits the generalisability of the study. Another limitation is that a convenience rather than a random sample was used consisting of persons seeking HIV testing.

The current study design does not include a control group, which makes it impossible to compare the prevalence of GAD among our sample with a comparison group of individuals not seeking HIV testing. However, it is necessary to note that the aim of this article was to report on the psychometric properties of the BAI and not to make definitive statements about the prevalence of anxiety. Further, the SCID and self-report questionnaires were not translated to Afrikaans and isiXhosa because of limited resources and time and thus only English-speaking participants were included in the study.

## Conclusion

Our findings demonstrate that the BAI may be used to screen for anxiety among HIV test seekers in South Africa. Nonetheless, a two-tiered approach is recommended to improve the problem of over-detection. In settings where resources are limited, the BAI may assist with the identification of those individuals in need of follow-up assessment and referrals for mental health treatment prior to or in conjunction with receipt of an HIV test result. In terms of further research, it is necessary to develop effective screening instruments and procedures that are more appropriate and feasible to implement in local settings, including among non-English speaking participants.

## References

[1] Adewuya AO, Afolabi MO, Ola BA, Ogundele OA, Ajibare AO, Oladipo BF Psychiatric disorders among the HIV-positive population in Nigeria: A control study. J Psychosom Res. 2007;63(2):203–206. 10.1016/j.jpsychores.2007.03.006 17662758

[2] Fincham D, Smit J, Carey P, Stein DJ, Seedat S The relationship between behavioural inhibition, anxiety disorders, depression and CD4 counts in HIV-positive adults: A cross-sectional controlled study. AIDS Care. 2008;20(10):1279–1283. 10.1080/09540120801927025 19012085

[3] Freeman M, Nkomo N, Kafaar Z, Kelly K Factors associated with prevalence of mental disorder in people living with HIV/AIDS in South Africa. AIDS Care. 2007;19(10):1201–1209. 10.1080/09540120701426482 18071963

[4] Hoffman DL, Dukes EM, Wittchen HU Human and economic burden of generalized anxiety disorder. Depress Anxiety. 2008;25(1):72–90. 10.1002/da.20257 17146763

[5] Ruscio AM, Hallion LS, Lim CC, et al Cross-sectional comparison of the epidemiology of DSM-5 generalized anxiety disorder across the globe. JAMA Psychiatry. 2017;74(5):465–475. 10.1001/jamapsychiatry.2017.0056 28297020 PMC5594751

[6] Strumpher J, Van Rooyen RM, Topper K, Andersson LM, Schierenbeck I Barriers to accessing mental health care in the Eastern Cape Province of South Africa. Afr J Nurs Midwifery. 2014;1(1):45–59. 10.25159/2520-5293/1487

[7] Zou KH, Liu A, Bandos AI, Ohno-Machado L, Rockette HE Statistical evaluation of diagnostic performance: Topics in ROC analysis. Boca Raton, FL: CRC Press; 2011 10.1111/insr.12020_27

[8] Wong HB, Lim GH Measures of diagnostic accuracy: Sensitivity, specificity, PPV and NPV. Proc Singapore Healthc. 2011;20(4):316–318. 10.1177/201010581102000411

[9] Coyne JC, Thompson R, Palmer SC, Kagee A, Maunsell E Should we screen for depression? Caveats and potential pitfalls. Appl Prev Psychol. 2000;9(2):101–121. 10.1016/S0962-1849(00)80009-8

[10] Kagee A, Tsai AC, Lund C, Tomlinson M Screening for common mental disorders in low resource settings: Reasons for caution and a way forward. Int Health. 2013;5(1):11–14. 10.1093/inthealth/ihs004 23580905 PMC3619733

[11] Van Den Heuvel L, Chishinga N, Kinyanda E, et al Frequency and correlates of anxiety and mood disorders among TB-and HIV-infected Zambians. AIDS Care. 2013;25(12):1527–1535. 10.1080/09540121.2013.793263 23668833

[12] Kagee A, Coetzee B, Saal W, Nel A Using the Beck anxiety inventory among South Africans living with HIV: Exploratory and higher order factor analyses. MECD. 2015;48(3):204–213. 10.1177/0748175615578734

[13] Mayston R, Patel V, Abas M, et al Symptoms of common mental disorder and cognitive associations with seropositivity among a cohort of people coming for testing for HIV/AIDS in Goa, India: A cross-sectional survey. BMC Public Health. 2013;13(1):204 10.1186/1471-2458-13-204 23497308 PMC3600001

[14] Saal WL Common mental and substance use disorders among people seeking HIV testing [dissertation]. Stellenbosch: Stellenbosch University; 2017 [cited 2019 October 9]. Available from: https://scholar.sun.ac.za/

[15] Shear MK, Greeno C, Kang J, et al Diagnosis of nonpsychotic patients in community clinics. Am J Psychiatry. 2000;157(4):581–587. 10.1176/appi.ajp.157.4.581 10739417

[16] Steiner JL, Tebes JK, Sledge WH, Walker ML A comparison of the structured clinical interview for DSM-III-R and clinical diagnoses. J Nerv Ment Dis. 1995;183(6):365–369. 10.1097/00005053-199506000-00003 7798084

[17] Kranzler HR, Tennen H, Babor TF, Kadden RM, Rounsaville BJ Validity of the longitudinal, expert, all data procedure for psychiatric diagnosis in patients with psychoactive substance use disorders. Drug Alcohol Depend. 1997;45(1):93–104. 10.1016/s0376-8716(97)01349-5 9179511

[18] Breuer E, Stoloff K, Myer L, Seedat S, Stein DJ, Joska JA The validity of the substance abuse and mental illness symptom screener (SAMISS) in people living with HIV/AIDS in primary HIV care in Cape Town, South Africa. AIDS Behav. 2014;18(6):1133–1141. 10.1007/s10461-014-0698-y 24452497

[19] Beidel DC, Frueh BC, editors Adult psychopathology and diagnosis Hoboken, NJ: John Wiley & Sons; 2018.

[20] Beck AT, Epstein N, Brown G, Steer RA An inventory for measuring clinical anxiety: Psychometric properties. J Consult Clin Psychol. 1988;56(6):893 10.1037//0022-006x.56.6.893 3204199

[21] Beck A, Steer R Manual of the Beck anxiety inventory. San Antonio, TX: The Psychology Corporation; 1990.

[22] Steele GI, Edwards DJ Development and validation of the Xhosa translations of the Beck inventories: 2. Item analysis, internal consistency and factor analysis. J Psychol Afr. 2008;18(2):217–226. 10.1080/14330237.2008.10820189

[23] Olley BO, Seedat S, Stein DJ Persistence of psychiatric disorders in a cohort of HIV/AIDS patients in South Africa: A 6-month follow-up study. J Psychosom Res. 2006;61(4):479–484. 10.1016/j.jpsychores.2006.03.010 17011355

[24] Swets JA Measuring the accuracy of diagnostic systems. Science. 1988;240(4857):1285–1293. 10.1126/science.3287615 3287615

[25] Eack SM, Singer JB, Greeno CG Screening for anxiety and depression in community mental health: The Beck anxiety and depression inventories. Community Ment Health J. 2008;44(6):465–474. 10.1007/s10597-008-9150-y 18516678

